# Premotor Symptoms as Predictors of Outcome in Parkinsons Disease: A Case-Control Study

**DOI:** 10.1371/journal.pone.0161271

**Published:** 2016-08-17

**Authors:** Yu-Hsuan Wu, Wei-Ju Lee, Yi-Huei Chen, Ming-Hong Chang, Ching-Heng Lin

**Affiliations:** 1 Section of Neurology, Taichung Veterans General Hospital, Taichung, Taiwan; 2 Department of Neurology, National Yang-Ming University School of Medicine, Taipei, Taiwan; 3 Department of Medical education and Research, Taichung Veterans General Hospital, Taichung, Taiwan; Oslo Universitetssykehus, NORWAY

## Abstract

**Background:**

To evaluate the association between the premotor symptoms and the prognosis of PD.

**Methods:**

A total of 1213 patients who were diagnosed of PD from January 2001 to December 2008 were selected from the Taiwan’s National Health Insurance Research Database. Patients were traced back to determine the presence of premotor symptoms, including rapid eye movement sleep behavior disorder (RBD), depression, and constipation. Cox’s regression analysis was used to detect the risks between the occurrence of premotor symptoms and the outcome (including death, psychosis, accidental injury, dementia and aspiration pneumonia). In addition, the association between premotor symptoms and levodopa equivalent dosage (LED) was examined.

**Results:**

Higher occurrence of death, dementia and aspiration pneumonia were identified in PD patients with premotor symptoms than without premotor symptoms (HR 1·69, 95% CI 1·34–2·14, p <0·001 for death; HR 1·63, 95% CI 1·20–2·22, p = 0·002 for dementia; HR 2·45, 95% CI 1·42–4·21, p = 0·001 for aspiration pneumonia). In a comorbidities-stratified analysis, PD patients with premotor symptoms showed significantly high risks of mortality and morbidity (dementia and aspiration pneumonia), especially in the absence of comorbidities. Independent predictors of mortality in PD were found to be higher age, male sex, constipation, RBD, RBD with constipation and depression, and diabetes. Furthermore, no significant differences of LED and subsequent accidental injury were noted between PD patient with or without premotor symptoms.

**Conclusion:**

Premotor symptoms seem to be not merely risk factors, but also prognostic factors of PD.

## Introduction

Survival in patients with Parkinson’s disease (PD) is reduced compared with the general population. Recent reports have revealed hazard ratios (HR) of mortality in PD patients ranging from 1·52 in a community-based Norwegian study to 3·38 in a door-to-door survey in Taiwan [1]. According to the University of Virginia Movement Disorders Database where the cause of death by clinical impression was determined for 197 of the 211 patients, about half of the patients died of PD-related causes, and the remainder of cardiovascular disease (12·3%), cancer (8·5%) and stroke (6·6%). Full autopsies were performed in ten PD patients. The majority of patients died of bronchopneumonia (five of ten patients) with one patient dying of complications related to a hip fracture. This indicated that 60% underwent full autopsy died of PD-related causes [2].

Higher age, male sex, severity of motor symptoms and gait dysfunction were found to be associated with increased mortality in patients with PD [3]. Non-motor symptoms are also increasing recognized as strong contributing factors of morbidity [4]. Dementia, the presence of psychotic symptoms and depression appear to be independent predictors of mortality in PD [3].

A growing number of articles have reported that certain non-motor symptoms might precede the development of motor symptoms in PD by several years. These premotor symptoms include rapid eye movement sleep behavior disorder (RBD), depression, and constipation. Although the role of premotor symptoms in predicting the subsequent development of PD is well understood [5], reports of the influence on disease progression and prognosis in PD of premotor symptoms are scarce.

Our study aimed to indentify whether the premotor symptoms can predict the prognosis in PD patients and also to investigate which of the premotor symptoms is the best predictor of subsequent prognosis in patients with PD.

## Methods

### Study design and data sources

Taiwan’s National Health Research Insurance (NHRI) program is a health insurance program that was instituted in 1995, and by 2010 it covered nearly 98% of all residents of Taiwan [6]. The National Health Insurance Research Database (NHIRD) contains comprehensive information regarding clinical visits and is managed by the Bureau of National Health Insurance. This study used as the data source the Longitudinal Health Insurance Database 2005, which consists of all comprehensive medical care coverage between 1996 and 2011 of a randomly selected group of beneficiaries (n = one million) in the 2005 Registry of Beneficiaries. The distribution of study subjects were representative of the national population in Taiwan.

### Standard protocol approvals, registrations and patients consents

The release of data was approved by the ethics committee of NHRI and the study protocol complied with the Declaration of Helsinki. Written consent from participants was not obtained because the NHI dataset were analyzed anonymously.

### Study population

Subjects with newly diagnosed PD from January 2001 to December 2008 were selected as the study group. The definition of PD was based on the International Classification of Diseases, 9^th^ revision and clinical modification (ICD-9-CM) code was 332·0 [7]. A previous validation study using a hospital administrative database reported a positive predictive value of more than 90% by using this definition of PD [8]. To further improve diagnostic validity, only subjects whose diagnostic code of PD was made in three or more consecutive visits at outpatient clinics or those diagnosed with PD during hospitalization were included as our PD subjects. We excluded patients who took anti-dopaminergic medicine (S1A Table) more than three times within three months before the diagnosis of PD. We also excluded the patients at risks for secondary or atypical Parkinsonism (S1B Table). Participants who had never taken any anti-parkinsonian medicine after the diagnoses of PD were also excluded.

### Premotor symptoms

The analyzed premotor symptoms included depression [9] (ICD-9-CM: 300·4, 309·0 occurring at least three or more times at outpatient clinics or more than once during hospitalization) and constipation (ICD-9-CM: 564·0 and receiving prescriptions with laxatives at least three times) [8]. Although the ICD-9 CM code for RBD is 327·42, it is not a familiar code for doctors in Taiwan. Therefore, to avoid underestimation, we also included the subjects who received clonazepam every night at least three times, but in the absence of insomnia and anxiety (ICD-9-CM: 307·41–42, 780·52, 780·54, 300·0, 293·84, 309·21). In our previous reports, the prevalence of idiopathic RBD in non-PD group was estimated at 1.2% [7]. It was compatible with the finding from a recent study using time-synchronized video-polysomnography [10]. The codes for premotor symptoms were extracted in the years before diagnosis of PD. PD patients with and without premotor symptoms were observed until death or on December 31, 2011.

### Confounders

Potential confounders included hypertension, diabetes mellitus (DM), hyperlipidemia, and ischemic heart disease (S1C Table). Ischemic heart disease was selected because it was found to the cause of mortality in PD patients. Hypertension, DM and hyperlipidemia were selected because these are risk factors for cardiovascular disease and stroke which were the common etiologies of mortality.

### Outcomes

The primary clinical outcome was mortality. Other associated prognostic factors were new-onset psychosis, accidental injury, dementia and aspiration pneumonia (S1D Table). The secondary clinical outcome was levodopa equivalent dosage (LED) of all anti-parkinsonian drugs at the end of every year after diagnoses of PD.

### Statistics

Stata version 11·1 was used to perform all statistical analyses. The PD patients were divided into two groups according to the presence and absence of the selected premotor symptoms before the diagnosis of PD. Student’s t test and Pearson chi-squared test were used to compare the age, the distributions of gender and confounders (hypertension, diabetes mellitus, hyperlipidemia, and ischemic heart disease) between the two groups of PD patients.

The crude incidence rate was calculated as the number of incident cases divided by the combined person-years from each individual in the cohort. A comparison of the risks of death, new-onset psychosis, accidental injury, dementia and aspiration pneumonia between the PD patients with premotor symptoms and PD patients without premotor symptoms were presented as crude hazard ratios (HRs). The adjusted HRs were estimated in a multivariate Cox’s proportional hazards model adjusting for age, gender, hypertension, diabetes mellitus, hyperlipidemia and ischemic heart disease.

To clarify the impact of confounders, the Cox models were also calculated separately for each of the following factors: hypertension, diabetes mellitus, hyperlipidemia, and ischemic heart disease. All variables of age, gender, and confounders with either single or multiple premotor symptoms were included in the regression analysis to predict the risk of death, dementia and aspiration pneumonia.

We also used Student’s t-test to analyze LED at the end of every year after diagnoses of PD in subjects with and without premotor symptoms. P values <0·05 or CIs for HRs that excluded the value 1·00 were considered statistically significant.

## Results

### Baseline cohort characteristics

Table 1 shows the demographic characteristics and co-morbidities of the study participants. A total of 1213 patients were newly diagnosed with PD, among whom 51·8% were male. The average age at the time of PD diagnosis was 62·9 ± 17·0 years. The patients who had premotor symptoms (n = 611) accounted for 50·4% of the patients with PD. The subjects with premotor symptoms were assigned as the study group and the patients without premotor symptoms were appointed as the control group. Within the study group, 36 patients had a history of RBD (5·9%), 211 patients had depression (34·5%) and 481 subjects had constipation (78·7%) before the diagnosis of PD. The detailed distribution of premotor symptoms is shown in S1E Table. In comparison with control group, the study group was on average older (64·0 ± 17·5 vs. 61·7 ± 16·5 years) and had a significantly higher occurrence of co-morbidities (including hypertension, diabetes, hyperlipidemia and ischemic heart disease) (p <0·05). The total mean follow-up time was 6·3 ± 2·6 years, but the mean follow-up time was shorter in the study group than in the control group (6·0 ± 2·6 years vs. 6·6 ± 2·6 years).

**Table 1:** Clinical characteristics of PD patients with and without premotor symptoms.

Variable	Total (N = 1213)	Without premotor symptoms (N = 602)	With premotor symptoms (N = 611)	p-value
	N (%)	N (%)	N (%)	
Age, years (mean±SD)	62·9±17·0	61·7±16·5	64·0±17·5	0·019 ^a^
Gender (male)	628 (51·8)	303(50·3)	325(53·2)	0·319
Hypertension ^b^	452 (37·3)	191(31·7)	261(42·7)	<0·001
Diabetes ^c^	202 (16·7)	74(12·3)	128(20·9)	<0·001
Hyperlipidemia ^d^	174(14·3)	74(12·3)	100(16·4)	0·043
Ischemic heart disease ^e^	179 (14·8)	66(11·0)	113(18·5)	<0·001

^a^ Student’s t test; chi-squared test for all other p-values.

^b^ Definition of hypertension: patients with diseases of the following ICD codes: 401–405 / A260,A269.

^c^ Definition of diabetes: patients with diseases of the following ICD codes: 250/ A181.

^d^ Definition of hyperlipidemia: patients with diseases of the following ICD codes: 272/ A182.

^e^ Definition of ischemic heart disease: patients with diseases of the following ICD codes: 410–414.

Comorbidities was defined as at least once clinical diagnosis within 1 year before PD first diagnosis date

PD: Parkinson’s disease, N: case number, %: percentage. SD: standard deviation.

### The hazard ratio of outcome variables associated with premotor symptoms

Results of outcome between the study group and control in terms of death, new-onset psychosis, dementia, accidental injury, and aspiration pneumonia are summarized in Table 2. The incident rate of death in study group (48·2 / 1000 person-years) was significantly higher than that in control patients (29·0 / 1000 person-years, p < 0·001). The incidence of dementia and aspiration pneumonia was significantly higher in the study group than in the control group (30·7 vs. 18·2 and 11·5 vs. 4·8 / 1000 person-years, p = 0·002 and p = 0·001, respectively). After adjusting for age, sex, and comorbidities, the study group still showed an increased risk for death, dementia and aspiration pneumonia with HRs of 1·40, 1·44 and 2·24, respectively, in comparison to the control group (p = 0·006 for death, p = 0·025 for dementia and p = 0·004 for aspiration pneumonia).

**Table 2:** Incidence and hazard ratio of outcome variables associated with premotor symptoms in Cox's regression analysis.

Outcome	Premotor symptoms						Crude HR (p-value)	Adjusted HR(95% CI)	p-value
	No (N = 602)			Yes (N = 611)					
	Event	Person-years	Incident rate (per1000 person-years)	Event	Person-years	Incident rate (per1000 person-years)			
Death	115	3960	29·0	178	3696	48·2	1·69 (<0·001)	1·40 (1·10–1·79)	0·006
Psychosis	8	3922	2·0	17	3617	4·7	2·20 (0·066)	2·25 (0·96–5·27)	0·060
Accidental injury	112	3409	32·9	117	3121	37·5	1·11 (0·415)	1·09 (0·84–1·42)	0·504
Dementia	67	3673	18·2	102	3320	30·7	1·63 (0·002)	1·44 (1·05–1·97)	0·025
Aspiration pneumonia	19	3922	4·8	42	3637	11·5	2·45 (0·001)	2·24 (1·29–3·90)	0·004

Adjusted HR was adjusted for Age, Gender, Hypertension, Diabetes, Hyperlipidemia, Ischemic heart disease.

HR: hazard ratio, CI: confidence interval, N: case number, %: percentage.

### The hazard ratio of outcome variables based on comorbidities

We provided the information about effects of premotor symptoms in the subgroups (according to comorbidities) of the study population on prognosis (S1F Table). The absence of diabetes, hyperlipidemia, and ischemic heart disease, yielded similar adjusted HRs (approximately 1·31–2·66, all p < 0·05) for subsequent death, psychosis, and dementia in the study group compared with control group. In hypertension-stratified analysis, adjusted HRs for death (HR 1·42, p = 0·032) and aspiration pneumonia (HR 2·12, p = 0·029) in non-hypertensive patients and for subsequent dementia (HR 1·63, p = 0·047) in hypertensive patients showed statistical significance. Moreover, the adjusted HRs for subsequent death, psychosis, and dementia in patients who had histories of comorbidities were mostly higher in PD patients with premotor symptoms, but the differences were not statistically significant.

### The hazard ratio of outcome variables associated with single or multiple premotor symptoms

The multivariate regression analysis (S1 Fig) showed that the variables which could predict prognosis.

### Age, sex and comorbidities

Higher age was associated with poor prognosis for PD (HR 1·07, 95% CI 1·06–1·08, p <0·001 for mortality, HR 1·08, 95% CI 1·06–1·09, p <0·001 for dementia, and HR 1·07, 95% CI 1·04–1·10, p <0·001 for aspiration pneumonia). Male sex was an independent factor for mortality (HR 1·49, 95% CI 1·17–1·91, p = 0·001). Diabetes was associated with a higher risk of mortality and dementia for PD (HR 1·49, 95% CI 1·12–1·97, p = 0·006 and HR 1·58, 95% CI 1·08–2·29, p = 0·017, respectively).

### Single premotor symptom

The roles of single premotor symptoms on prognosis for PD were not consistent among the three premotor symptoms. Constipation significantly increased mortality and morbidities for PD (HR 1·45, 95% CI 1·12–1·87, p = 0·005 for mortality; HR 1·51, 95% CI 1·08–2·12, p = 0·017 for dementia; HR 2·59, 95% CI 1·44–4·65, p = 0·002 for aspiration pneumonia). However, neither RBD nor depression had a significant effect on prognosis, except for RBD on mortality in PD (HR 2·71, 95% CI 1·10–6·65, p = 0·030).

### Multiple premotor symptoms

Higher mortality and morbidity was noted when RBD was added to constipation or/and depression (for mortality, HR 4·75, 95% CI 1·47–15·36, p = 0·009 in patients combining RBD with depression and constipation; for dementia, HR 8·05, 95% CI 1·94–33·39, p = 0·004 in patients combining RBD with depression).

### The levodopa equivalent dosage (LED) with premotor symptoms

In the first five years, a significantly higher LED in the study group than in the control group was only noted in the initial two years (292·2±193·6 mg/day in study group, 261·1±164·4 mg/day in control group, p = 0·042 in the first year; 354·2±257·5 mg/day in study group, 299·4±199·2 mg/day in control group; p = 0·012 in the second year). However, the tendency of a higher LED in study group diminished gradually thereafter (553·4±456·4 mg/day in control group, 494·6±347·9 mg/day in study group, p = 0·355 in end of 8–11 ^th^ year).

## Discussion

There are two main findings of the current study: 1) PD patients with premotor symptoms (including RBD, constipation, and depression) were associated with higher risks for death, dementia and aspiration pneumonia, especially in absence of comorbidities (diabetes, hyperlipidemia and ischemic heart disease); 2) older age, male sex, constipation, RBD, RBD with constipation and depression, and diabetes were independent predictors of mortality in PD patients.

According to previous 10-yearlongitudinal study, the most frequent cause of death in PD was pneurmonia (30%), followed by cardiovascular disease (21%) and cancer (19%) [11].Our results were consistent with these findings and demonstrated increased mortality in PD patients with old age, male sex, history of diabetes and presence of premotor symptoms. It is reasonable that old age, male sex and diabetes are strong independent risk factors for cardiovascular events. We further identified that premotor symptoms are independent prognostic factors, especially when PD patients had no underlying disease such as diabetes, ischemic heart disease, hypertension and hyperlipidemia. The higher risk of aspiration pneumonia in PD patients with premotor symptoms may be a possible association with increased mortality.

RBD appeared to have significant effects on prognosis, including death and dementia. Several studies suggested PD patients with RBD may have worse prognosis in terms of impaired cognitive function and overall morbidity/mortality than PD patients without RBD [12]. One study revealed the most consistent links between RBD and PD were orthostatic hypotension and higher frequency of freezing. There were also probable associations between RBD and depression, akinetic-rigid subtype and falls. This suggests that PD with RBD may result from a different pattern of neurodegenerative process than PD without RBD [13].

Constipation, as a part of autonomic dysfunction, showed poor prognostic effect on death, dementia and aspiration pneumonia. One study revealed the density of submucosal Lewy neurites was significant correlated with axial scores and the severity of constipation. The associations were not found with disease duration and motor symptoms. Furthermore, it suggested that dysautonomic, axial and cognitive symptoms characterized disease severity [14]. The similar relationship between constipation and prognosis from our study lend additional support to the previous findings. However, the association between increased mortality/morbidities and autonomic dysfunction remains unclear.

We indentified that PD patients with premotor symptoms were older at PD onset and had significantly higher occurrence of co-morbidities. It has been noted that patients presenting with non-motor symptoms of PD were frequently misdiagnosed initially by primary physicians, leading to delayed diagnosis of PD, with a median interval of 1.6 years [15]; this may at least in part explain why age of PD onset in PD patients with premotor symptoms was higher than controls. The reason for higher the occurrence of co-morbidities in PD patients with premotor symptoms requires elucidation, but may be associated with older age.

The LED in our study was relatively smaller than that reported in studies from Europe and America [3,16]. According to other reports from Asian populations, the LED were relatively small (Japanese patients 419·5 mg/day) [17]. Thus, ethnic differences may exist in the LED in PD patients.

A higher LED possibly reflects more severe motor impairment. In our report, initially higher LED in PD patients with premotor symptoms may have resulted from worse motor condition. We suppose that the initially higher LED in study group may be associated with delayed diagnosis or more confounding factors, though the trend of a higher LED diminished gradually thereafter. Our reports also showed no significant difference of following accidental injuries between PD patients with or without premotor symptoms. It seems that there was no clear association between premotor symptoms and outcome of motor impairment in PD patients.

Whether the premotor symptoms are independent prognostic factors for PD remains unclear due to the sparse literature. Our population-based, retrospective study found a higher risk of mortality, dementia, and aspiration pneumonia in PD patients with premotor symptoms than in control subjects, especially in the absence of comorbidities. Constipation was an independent and poor prognostic factor for PD. The role of RBD for PD patients had a synergic effect on prognosis: PD patients with a history of RBD combined with either depression or constipation may have a poorer prognosis. These findings suggest that premotor symptoms are not just risk factors but prognostic factors of PD. Moreover, due to the lack of significant differences for LED and subsequent accidental injury between PD patients with or without premotor symptoms, we assume that the premotor symptoms are associated with non-motor symptoms after PD onset, but possibly not in motor symptoms.

The strengths of the present study are its large sample size and longitudinal follow-ups with a long duration. In addition, because participation in the NHI is mandatory and easily accessible with low payment, follow-up compliance is high. In Taiwan, most patients (82.9%) received their first anti-PD medication in referred hospitals. About half of PD patients received their initial prescription from neurologists [18].There are also some limitations of our study. First, our cross-sectional study design only demonstrated associations between premotor symptoms and prognosis in PD patients rather than a causal relationship. Second, we could not obtain the clinical review of patient data from the NHIRD. Therefore, we could not completely exclude the patients with secondary parkinsonism or atypical parkinsonism in our study groups. Furthermore, other potential factors contributing to prognosis of PD, e.g., Hoehn and Yahr stage, causes of death, severity of premotor symptoms, formal autonomic function tests are not available in the NHIRD. Third, the NHIRD only allows us to follow data for less than 11 years. Because the survival of PD could extend to more than 15 years, the study group in our report may be restricted in terms of its representativeness of the PD population. Fourth limitation is the diagnosis of RBD. According to criteria of International Classification of Sleep Disorders (ICSD-2), RBD should be diagnosed based on polysomnography, history and absence of EEG epileptiform activity during REM sleep. The definition of RBD in our study might be over-estimated and not very accurate. Fifth, we did not include all participants in NHIRD for analysis. However, a prior study showed that the distributions of the selected sampling were representative of the Taiwanese population [19]. Even though, we could not present the detailed reports due to the weakness of ICD codes, the trends found from our study may still provide some important information.

## Supporting Information

S1 Fig Hazard ratio of outcome variables associated with premotor symptoms in Cox’s regression analysis. (TIF)

S1 Table Drugs with high risk of extrapyramidal symptoms (**S1A Table)**. Diseases with risk of secondary or atypical Parkinsonism(**S1B Table**). Potential confounders **(S1C Table).** Primary clinical outcomes(**S1D Table).** Distribution of premotor symptoms (**S1E Table).** Hazard ratio associated with premotor symptoms in Cox's regression analysis, as stratified by co-morbidities **(S1F Table)**.
(DOC)
