# Supplementary figures and images for: Premotor Symptoms as Predictors of Outcome in Parkinsons Disease: A Case-Control Study
Source: PLoS One. 2016 Aug 17;11(8):e0161271. doi: 10.1371/journal.pone.0161271 (PMC4988705; doi:10.1371/journal.pone.0161271)

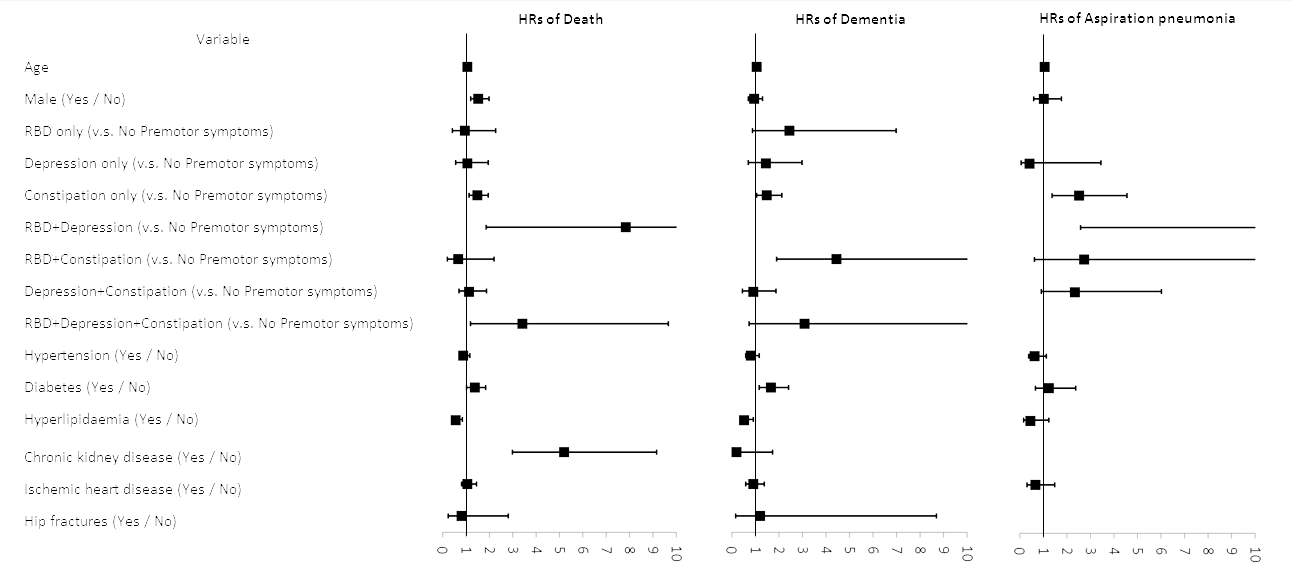

Supplement: S1 Fig — (TIF) [file pone.0161271.s001.tif]
