# Supplementary material for: Premotor Symptoms as Predictors of Outcome in Parkinsons Disease: A Case-Control Study
Source: PLoS One. 2016 Aug 17;11(8):e0161271. doi: 10.1371/journal.pone.0161271 (PMC4988705; doi:10.1371/journal.pone.0161271)
Supplement: S1 Table — Drugs with high risk of extrapyramidal symptoms (S1A Table). Diseases with risk of secondary or atypical Parkinsonism(S1B Table). Potential confounders (S1C Table). Primary clinical outcomes(S1D Table). Distribution of premotor symptoms (S1E Table). Hazard ratio associated with premotor symptoms in Cox's regression analysis, as stratified by co-morbidities (S1F Table). (DOC) [file pone.0161271.s002.doc]

**Table A. Drugs with high risk of extrapyramidal symptoms**

| **Drug category** | **Generic drugs** |
| --- | --- |
| Anti-emetics | Metoclopramide, Prochlorperazine |
| Neuroleptics | Haloperidol, Amisulpride, Flupentixol, Fluphenazine, Levomepromazine, Pimozide, Amisulpride, Thioridazine, Zuclopenthixol, Risperidone, Olanzapine, Aripiprazole |
| Calcium channel blocker | Flunarizine, Cinnarizine |
| Dopamine depleter | Reserpine, Tetrabenazine |
| Dopamine synthesis blocker | Methyldopa |

**Table B. Diseases with risk of secondary or atypical Parkinsonism**

| **Diseases** | **ICD-9-CM** |
| --- | --- |
| Stroke | 430-438/A290-A294,A299 |
| Dementia | 290,331.0, 331.2/A210 |
| Meningitis, encephalities | 00321,﻿0065,﻿0130-3,﻿0136,﻿0360-1,﻿0460-3,﻿047,﻿0490-1,0520,﻿0530,﻿0543,﻿05472,﻿0550,﻿05601,﻿062-4,﻿0721-2,﻿09041-2,﻿0941-2,﻿09481-2,﻿09487,﻿09882,﻿10081,﻿11283,﻿1142,﻿11501,﻿11511,﻿11591,﻿1300,﻿1390,﻿320-6 |
| Head injury | 800-1, 803-4, 850-4 |
| Hydrocephalus | 742.3, 741.0, 331.3-4 |
| Brain tumor | 191, 192.0-1, 192.8-9, 194.3-4, 198.3, 237.0-1, 237.5-6, 237.9, 239.6-7, 2250, 2252, ﻿2273-4, ﻿22802 |
| Congenital or hereditary disorders | 2750-1,﻿ 3334,﻿ 334, ﻿740 |
| Hypoxic encephalopathy | 348.1, 997.01, 639.8, 669.4, 768.7, 779.2 |

**Table C. Potential confounders**

| **Diseases** | **ICD-9-CM** |
| --- | --- |
| Hypertension (HTN) | 401-405 / A260,A269 |
| Diabetes (DM) | 250/ A181 |
| Hyperlipidemia | 272/ A182 |
| Coronary artery disease | 410-414 |

**Table D. Primary clinical outcomes**

| **Diseases** | **ICD-9-CM** |
| --- | --- |
| Psychosis | 780.1,292,292.10-2,293.81-2, 297,298.3-4,301.0 |
| Accidental injury^1^ | head injuries: 800–804, 850–854 and 959.01, A470,A490-1  bone fractures and dislocations: 805–808, 810–828 and 831–839  burns: 940–949  injuries to the spinal cord, plexus and nerves: 767.4, 767.6 and 952–957  superficial injuries and contusions: 910–924 |
| Dementia | 290,﻿331.0,331.2,A210 |
| Aspiration pneumonia | 482, but exclude 482.84 |

e-Reference:

1. Wang HC, Lin CC, Lau CI, Chang A, Sung FC, Kao CH. Risk of accidental injuries amongst Parkinson disease patients. Eur J Neurol. 2014 Jun;21(6):907-13.

**Table E. Distribution of premotor symptoms**

| **Variables** | **With premotor symptoms (N=611) N (%)** |
| --- | --- |
| RBD only | 18 (2·9) |
| Depression only | 109 (17·8) |
| Constipation only | 375 (61·4) |
| RBD and depression | 3 (0·5) |
| RBD and constipation | 7 (1·1) |
| Depression and constipation | 91 (1·9) |
| RBD, depression and constipation | 8 (1·3) |

RBD: rapid eye movement behavior disorder, %: percentage

**Table F. Hazard ratio** associated with premotor symptoms in Cox's regression analysis, as stratified by co-morbidities

| outcomes | | Crude HR(p-value) | Adjusted HR(p-value) | (95% CI) |
| --- | --- | --- | --- | --- |
| **Hypertension** | | | |  |
| No | Death | 1·57 (0·005) | 1·42 (0·032) | (1·03-1·97) |
|  | Dementia | 1·37 (0·141) | 1·41 (0·112) | (0·92-2·16) |
|  | Aspiration pneumonia | 2·17 (0·022) | 2·12 (0·029) | (1·08-4·16) |
| Yes | Death | 1·61 (0·009) | 1·38 (0·081) | (0·96-1·98) |
|  | Dementia | 1·83 (0·014) | 1·63 (0·047) | (1·01-2·65) |
|  | Aspiration pneumonia | 2·96 (0·031) | 2·51 (0·070) | (0·93-6·78) |
| **Diabetes** | | | |  |
| No | Death | 1·55 (0·001) | 1·35 (0·030) | (1·30-1·77) |
|  | Dementia | 1·59 (0·010) | 1·55 (0·018) | (1·08-2·22) |
|  | Aspiration pneumonia | 2·40 (0·004) | 2·30 (0·008) | (1·25-4·24) |
| Yes | Death | 1·90 (0·020) | 1·64 (0·074) | (0·95-2·82) |
|  | Dementia | 1·41 (0·302) | 1·27 (0·484) | (0·65-2·45) |
|  | Aspiration pneumonia | 2·37 (0·192) | 2·19 (0·242) | (0·59-8·19) |
| **Hyperlipidemia** | | | |  |
| No | Death | 1·67 (<0·001) | 1·36 (0·016) | (1·06-1·76) |
|  | Dementia | 1·58 (0·006) | 1·43 (0·038) | (1·02-1·99) |
|  | Aspiration pneumonia | 2·93 (<0·001) | 2·66 (0·001) | (1·47-4·81) |
| Yes | Death | 2·18 (0·058) | 1·91 (0·118) | (0·85-4·32) |
|  | Dementia | 2·35 (0·097) | 2·19 (0·133) | (0·79-6·09) |
|  | Aspiration pneumonia | 0·51 (0·466) | 0·42 (0·371) | (0·06-2·83) |
| **Ischemic heart disease** | | | |  |
| No | Death | 1·54 (0·001) | 1·31 (0·044) | (1·01-1·71) |
|  | Dementia | 1·63 (0·005) | 1·49 (0·024) | (1·06-2·10) |
|  | Aspiration pneumonia | 2·32 (0·004) | 2·15 (0·010) | (1·21-3·85) |
| Yes | Death | 2·23 (0·011) | 1·86 (0·054) | (0·99-3·50) |
|  | Dementia | 1·49 (0·322) | 1·34 (0·478) | (0·60-2·97) |
|  | Aspiration pneumonia | 4·83 (0·141) | 3·67 (0·231) | (0·44-30·71) |

Adjusted HR was adjusted for Age, Gender, Hypertension, Diabetes, Hyperlipidemia, Ischemic heart disease.

HR: hazard ratio, CI: confidence interval, %: percentage.
